# Flexibility in Faculty Work-Life Policies at Medical Schools in the Big Ten Conference: A Ten-Year Follow-up Study

**DOI:** 10.1089/whr.2021.0070

**Published:** 2022-01-31

**Authors:** Emily A. Wagner, Jaclyn H. Jansen, Hannah DeLuna, Katherine Anderson, Marla C. Doehring, Julie L. Welch

**Affiliations:** ^1^ Department of Emergency Medicine, Regions Hospital, HealthPartners; Department of Pediatrics, University of Minnesota, Minneapolis, Minnesota, USA.; ^2^ Department of Emergency Medicine, Indiana University School of Medicine, Indianapolis, Cincinatti, Ohio, USA.; ^3^ Department of Emergency Medicine, Indiana University School of Medicine, Indianapolis, Indiana, USA.

**Keywords:** work-life, wellness, burnout, gender equity, lactation support, academic employment

## Abstract

**Background:**

Flexible work-life policies for medical school faculty are necessary to support career progress, advancement, retention, and job satisfaction.

**Objective:**

Our objective was to perform a 10-year follow-up descriptive assessment of the availability of flexible work-life policies for faculty in medical schools in the Big Ten Conference.

**Design:**

In this descriptive study, a modified objective scoring system was used to evaluate the flexibility of faculty work-life policies at 13 medical schools in the Big Ten Conference. Policy information was obtained from institutional websites and verified with the human resources offices. Scores from the 2011 study and 2020 were compared.

**Results:**

Michigan State and Ohio State Universities offered the most flexible policies (score 17.75/22) with the University of Michigan following (score 16.75/22). The largest delta scores, indicating more flexible policies in the past decade, were at University of Michigan (6) and University of Minnesota (5.25). Policies for parental leave and part-time faculty varied widely. Most schools earned an additional point in the newly added category of “flexible scheduling and return-to-work policies.” Nearly every institution reported dedicated lactation spaces and improved childcare options.

**Limitations:**

Limitations included missing policy data and interpretation bias in reviewing the policy websites, unavailable baseline data for schools that joined the Big Ten after the 2011 study, and unavailable baseline data for the additional category of return-to-work policies.

**Conclusions:**

While progress has been made, every institution should challenge themselves to review flexibility in work-life policies for faculty. It is important to advance a healthy competition with the goal to achieve more forward-thinking policies that improve retention, recruitment, and advancement of faculty. Big Ten institutions can continue to advance their policies by providing greater ease of access to options, further expansion of parental leave and childcare support, and offering more flexible policies for part-time faculty.

## Introduction

Work-life balance is defined as the way in which an individual divides time between work and activities outside of work. Work-life well-being is becoming increasingly important to both employers and employees as millennials enter the workplace. The ability to recruit and retain a highly skilled and trained workforce is directly impacted by the employment policies of an institution.

Organizational policies that support the individual employee's personal health and wellness while also allowing pursuit of a rewarding, upwardly mobile career may contribute to greater job productivity while decreasing burnout.^1–5^ Monetary compensation, paid time off, flexible scheduling, childcare and backup childcare resources, on-site fitness facilities, healthy on-site dining options, and paid and unpaid leave policies are examples of ways, in which employers have tangibly supported work-life balance.^6^

Historically, the field of medicine has embraced a “live to work” mentality with its associated expectation of sacrificing individual wellness in the pursuit of clinical excellence.^7^ In recent years, many younger physicians have embraced a “work to live” mindset which has challenged leaders in academic medicine to creatively develop employment policies that promote a healthier balance between meaningful work and the physical, mental, and emotional health of individual physicians. This wide array of inclusive policies that allow for the enhancement of work-life integration is often defined as flexibility in the workplace.

While enrollment in medical school is now equal between genders,^8^ addressing the gender gap in faculty recruitment, promotion, and leadership positions has been slower and more challenging. Workplace flexibility is a primary concern for female professionals who continue to bear disproportionate responsibility for domestic work.^9,10^ In addition, the increasing prevalence of dual career couples among highly educated professionals along with rising expectations for fathers' involvement in childcare make work-life balance a challenge for a growing proportion of men.^11^ Improving work-life policies for academic physicians may minimize turnover for both men and women while decreasing the gender gap in academic advancement.

Over the last decades, increasing numbers of women have chosen careers in academic medicine. Some research efforts have highlighted problematic issues in work-life balance while others contributed to the development of creative policies, which facilitate combining successful careers with rewarding lives outside of medicine. For example, female physicians report improved job satisfaction when they do not delay important life events such as marriage or childbearing.^12^

In addition, studies by Clem et al. and Rizvi et al. have shown that career satisfaction is directly linked to flexible scheduling, supportive colleagues, and time spent with family.^13,14^ Another example of work-life balance advocacy by younger physicians was the successful implementation of flexible scheduling policies for pregnant residents.^15^

Both academic physicians and the universities that employ them can benefit from increased attention to work-life balance issues. When universities adopt policies that meet the needs of a younger, more diverse faculty, they are more likely to recruit, retain, and promote these talented individuals.

Universities can learn from forward-thinking employers in private industry that have successfully implemented initiatives that support both traditional and nontraditional gender roles, dual-career couples, and those who are caregivers for young children and/or aging relatives.^6^ Many physicians in these groups have historically been forced to choose between career advancement and providing care to their loved ones, which has contributed to a significant gender gap in academic advancement and promotion of female faculty in medicine.^9^

In 2008 and 2011, two independent studies were published that assessed employment policies affecting work-life balance for academic faculty in United States medical schools. Bristol et al. evaluated employment policies of the top 10 medical schools in the United States, while Welch et al. analyzed them among medical schools in the Big Ten Conference.^16,17^ The objective of our study was to perform a 10-year follow-up descriptive assessment of the Welch et al. study of the availability of flexible work-life policies for faculty in medical schools in the Big Ten Conference.

## Materials and Methods

### Study design and population

This study was a descriptive assessment of faculty work-life policies at medical schools in the Big Ten Conference in 2020. We followed a standard protocol similar to that described Bristol et al. and Welch et al.^16,17^ The findings were then compared to findings from the 2011 study to report any notable changes.^17^ As of 2020, 14 institutions comprise the Big Ten Conference as designated by the National Collegiate Athletic Association (NCAA). With the exception of Northwestern, which is a private/nonsectarian university of 22,000 students, the institutions are public universities with undergraduate enrollment ranging from 25,820 to 61,170 students.

Thirteen Big Ten universities with medical schools were included in this study: University of Illinois, Indiana University, University of Iowa, University of Maryland, University of Michigan, Michigan State University, University of Minnesota, University of Nebraska-Lincoln, Northwestern University, Ohio State University, Pennsylvania State University, Rutgers University, and the University of Wisconsin-Madison.

Purdue University does not have a medical school. Since the 2011 study, three of these institutions (Maryland, Nebraska, and Rutgers) were added to the Big Ten Conference, thus not included in the Welch et al. study.^17^

### Procedure

We accessed each institution's website between July 2020 and December 2020 and searched for information on policies for medical school faculty in the following categories: parental (maternity, paternity, and adoption) leave, extension of the tenure probationary period (defined as the time for the review before tenure), part-time appointments, part-time tenure track, health benefits for part-time employees, child-care options, including on campus care as well as subsidization, and lactation policies.

In addition, in this 10-year follow-up study, we added a category for postpartum scheduling (*i.e.*, prolonged flexible scheduling and return to work options in the postpartum and postadoption periods). Regarding part-time appointments, part-time tenure track, and part-time benefits, we used each institution's own definition of part-time when relevant.

### Search strategy

Each institution's website was accessed for medical school and/or parent university faculty policies *via* the human resources or benefits page. Once located, the search strategy terms included “faculty policies,” “leave policies,” “maternity leave,” “paternity leave,” “adoptive leave,” “flexible-scheduling,” “return to work,” “lactation,” “childcare,” “leave of absence,” “part-time,” “tenure,” “human resources,” “faculty handbook,” or the name of each policy category. The policy data were recorded into an excel spreadsheet and a score was assigned by one author. A second author independently reviewed the data and score, with discrepancies resolved by consensus.

### Verification of policies

To verify the accuracy of the work-life policy information gathered from the institution's website, an email was sent to each respective institution's Human Resource (HR) office contact person(s). In the email, we described the study, attached a summary of the policy information gathered from their website, and requested a phone or video call to verify their policies. Scores were adjusted based on information provided by the HR representative. If there was no email response, we sent two follow-up emails over 8 weeks. Upon completion on data collection, two authors again reviewed each institution's website for any updated policies to ensure all scores were the most current available. For institutions that failed to respond, scores represent information from online data collection only.

### Scoring and analysis

For an individual institution, each policy was scored on a scale as described in the Work-Life Policy Scoring Assessment, with higher scores indicating greater flexibility (Table 1).^16,17^ Two authors applied the scoring rubric and discrepancies were resolved by a third author. This scoring system was modified from Welch et al., which was based on Bristol et al., to include one additional policy of return-to-work scheduling. Category scores ranged from 0 to 3. Higher scores in each category indicated more flexible policies.

**Table 1.** Work-Life Policy Scoring Assessment

Policy category	Scoring definition
ML	0 FMLA 1 FMLA and accrued sick/disability payment 2 6–8 weeks paid leave 3 > 8 weeks paid leave Category average score applied to total score = [ML from day 1 of hire (0–3) ± ML after day 1 of hire (0–3) ± ML after 2 years of hire (0–3) ± ML for part-time faculty (0–3)]/4. Total average category score available, 0–3.
PL	0 FMLA 1 FMLA and accrued sick/vacation payment 2 < 4 weeks paid leave 3 > 4 weeks paid leave Category score applied to total score = [PL from day 1 of hire (0–3) ± PL after day 1 of hire (0–3) ± PL after 2 years of hire (0–3) ± PL for part-time faculty (0–3)]/4. Total category score available, 0–3.
AL	0 FMLA 1 FMLA and accrued sick/vacation payment 2 3–8 weeks paid leave 3 > 8 weeks paid leave Category score applied to total score = [AL from day 1 of hire (0–3) ± AL after day 1 of hire (0–3) ± AL after 2 years of hire (0–3) ± AL for part-time faculty (0–3)]/4. Total category score available, 0–3.
Extension of probationary period	0 < 1 year 1 1 year extension on request 2 1 year automatic extension 3 No probationary period
Part-time appointments	0 No part-time appointments available 1 Part-time appointments for special circumstances OR with time limit 2 Part-time appointments with no time limit
Part-time tenure track	0 No part-time tenure track 1 Offer part-time tenure track
Part-time health benefits	0 No part-time benefits offered 1 Benefits offered proportional to FTE> or = 75% 2 Benefits offered to proportional to FTE> or = 50% 3 Benefits offered proportional to FTE <50%
Child care options	0 No direct affiliation with child care centers 1 Affiliated child care centers available with no financial aid or discounts 2 Affiliated child care centers available with financial aid or discounts or both
Lactation policy	0 General policy with no online listing of dedicated space for breast pumping 1 Listing of dedicated spaces available for breast pumping
Return-to-work policy^a^	0 No policy regarding flexible return to work scheduling after the addition of a child 1 Policy for flexible scheduling with return to work or work conditions with the addition of a child
Total score without return-to-work policy: 21 Total score possible with return-to-work policy: 22	

Adapted and modified from Bristol et al. and Welch et al.^16,17^

^a^ New category added.

AL, adoption leave; FMLA, Family and Medical Leave Act; FTE, Full-time equivalent; ML, maternity leave; PL, paternity leave.

To address the variability of family leave policies available and their utility over the course of a career, these leave policies were further evaluated as to their availability on day of hire, after 1 year of employment, after 2 years of employment, and for part-time employees. An average score was then determined for each of the family leave policy categories (maternity, paternity, and adoptive leaves) with the maximum score of three available for each category. This expanded family leave scoring system considered varying family leave options. The average score ensured that it was not weighted more than the other policies measured in the total score.

A total score was then derived and assigned as the score for all policies for each institution (ranging from 0 to 21). A total score of 0–22 was also included to reflect the additional category of return-to-work scheduling.

To report changes in faculty policies in medical schools in the Big Ten between the 2011 study^16^ and 2020, a delta score was calculated by subtracting the total score in the 2011 study from the total score in 2020.

## Results

Work-life policy data were collected from their websites for all institutions with medical schools. An HR contact person was identified at each institution and asked to review each posted policy and verify accuracy. Data were verified for 5 of 13 schools (University of Illinois, Indiana University, Northwestern University, Ohio State University, and Pennsylvania State University). Other institutions referred us back to their websites without specific verification of collected scores.

Overall, more institutions possessed work-life websites in comparison to the 2011 study; however, websites were challenging to navigate, and obtaining data was time consuming. Most websites did not have a central repository of faculty policies, requiring searches of multiple web pages or online documents for each institution.

Michigan State University and Ohio State University demonstrated the most forward-thinking and complete work-life policies with a tied score of 17.75 cumulatively with the additional return-to-work category considered. Both universities earned a score of 6.75 for family leave policies specifically and scored additional points in part-time employment policies, lactation accommodations, childcare options, and return to work scheduling (Table 2).

**Table 2.** Total Scores for Faculty Work-Life Policy Assessment at Big Ten University Medical Schools

Policy: Total Score	Michigan State University	Ohio State University	University of Michigan	University of Minnesota	University of Maryland	Penn State University	University of Wisconsin	University of Nebraska	Northwestern University	Indiana University	University of Iowa	University of Illinois	Rutgers University
Family leave policies average (0–9)	6.75	6.75	5.75	7	6.75	4.5	2.25	3	4.5	5.25	2.25	2.25	0
Extension of probationary period (0–3)	2	1	2	1	0	0	1	3	2	2	2	2	0
Part-time appointments (0–2)	2	2	2	2	2	2	2	2	1	1	2	1	1
Part-time tenure track (0–1)	1	1	1	1	0	0	1	0	0	0	1	1	0
Part-time health benefits (0–3)	2	3	2	2	2	3	3	2	2	1	2	2	0
Child care options (0–2)	2	2	2	1	2	2	2	1	2	1	2	2	2
Lactation policy (0–1)	1	1	1	1	1	1	1	1	1	1	1	0	1
**Total score (0–21)**	**16.75**	**16.75**	**15.75**	**15**	**13.75**	**12.5**	**12.25**	**12**	**12.5**	**11.25**	**12.25**	**10.25**	**4**
Additional 2020 category: Return to work policy (0–1)	1	1	1	1	1	1	1	1	0	1	0	1	1
**Total score with additional category (0–22)**	**17.75**	**17.75**	**16.75**	**16**	**14.75**	**13.5**	**13.25**	**13**	**12.5**	**12.25**	**12.25**	**11.25**	**5**

In descending order after Michigan State and Ohio State Universities, the University of Michigan earned a cumulative score of 16.75, followed by the University of Minnesota (16), University of Maryland (14.75), Penn State University (13.5), University of Wisconsin (13.25), University of Nebraska (13), Northwestern University (12.5), Indiana University (12.25), University of Iowa (12.25), University of Illinois (11.25), and lastly, Rutgers University with a score of 5 (Table 2).

In comparison with the 2011 study, the greatest improvement was demonstrated by the University of Michigan, whose score improved from 9.75 to 15.75 with a delta score of 6 through significant improvement in parental leave policies. The University of Minnesota demonstrated the next greatest change in score from 9.75 to 15 with a delta score of 5.25 by improving leave policies, adding lactation policies, and increasing part-time benefits. The University of Wisconsin demonstrated no advancement in policies from the 2011 study to 2020 with an unchanged score of 12.25 (Table 3).

**Table 3.** Total Change in Score from 2011 to 2020 for Faculty Work-Life Policy Assessment at Big Ten University Medical Schools

Policy: 2011 v 2020 Total Score Change	University of Michigan	University of Minnesota	Michigan State University	Ohio State University	Northwestern University	Indiana University	University of Iowa	Penn State University	University of Illinois	University of Wisconsin	University of Maryland	University of Nebraska	Rutgers University
2011 (0–21)	9.75	9.75	13.25	13.5	9.5	9.25	11.5	12	10	12.25	N/A	N/A	N/A
2020 (0–21)	15.75	15	16.75	16.75	12.5	11.25	12.25	12.5	10.25	12.25	13.75	**12**	**4**
**Delta score**	**6**	**5.25**	**3.5**	**3.25**	**3**	**2**	**0.75**	**0.5**	**0.25**	**0**	**N/A**	**N/A**	**N/A**

Notably, Rutgers University earned the lowest score of 5 demonstrating minimal leave policies or part-time options (Table 2).

### Family leave policies

Availability of family leave policies varied between institutions. Scores ranged from 0 out of 9 at Rutgers University to 7 out of 9 at the University of Minnesota. The University of Minnesota demonstrates a robust leave policy offering leave availability from day one of hire for new birth mothers, new fathers, and new adoptive parents of both sexes. On average, institutions earned a score of 4.38. Compared to the average score of 3.18 in the 2011 study reported by Welch et al., policies regarding family leave have improved (Table 4).

**Table 4.** Expanded Family Leave Policy Scores for Faculty at Big Ten University Medical Schools

Policy: family leave	University of Minnesota	Ohio State University	University of Maryland	Michigan State University	University of Michigan	Indiana University	Northwestern University	Penn State University	University of Nebraska	University of Wisconsin	University of Iowa	University of Illinois	Rutgers University
Maternity leave from day 1 of hire (0–3)	2	1	0	0	2	0	0	0	0	0	0	0	0
Maternity leave after 1 year of hire (0–3)	2	2	3	3	2	2	3	3	2	1	1	1	0
Maternity leave after 2 years of hire (0–3)	2	2	3	3	2	2	3	3	2	1	1	1	0
Maternity leave for part-time faculty (0–3)	2	2	3	3	2	2	0	0	2	1	1	1	0
Maternity leave category average (0–3)^a^	2	1.75	2.25	2.25	2	1.5	1.5	1.5	1.5	0.75	0.75	0.75	0
Paternity leave from day 1 of hire (0–3)	3	1	0	0	0	0	0	0	0	0	0	0	0
Paternity leave after 1 year of hire (0–3)	3	3	3	3	3	3	3	3	1	1	1	1	0
Paternity leave after 2 years of hire (0–3)	3	3	3	3	3	3	3	3	1	1	1	1	0
Paternity leave for part-time faculty (0–3)	3	3	3	3	3	3	0	0	1	1	1	1	0
Paternity leave category average (0–3)^a^	3	2.5	2.25	2.25	2.25	2.25	1.5	1.5	0.75	0.75	0.75	0.75	0
Adoption leave from day 1 of hire (0–3)	2	1	0	0	0	0	0	0	0	0	0	0	0
Adoption leave after 1 year of hire (0–3)	2	3	3	3	2	2	3	3	1	1	1	1	0
Adoption leave after 2 years of hire (0–3)	2	3	3	3	2	2	3	3	1	1	1	1	0
Adoption leave for part-time faculty (0–3)	2	3	3	3	2	2	0	0	1	1	1	1	0
Adoption leave category average (0–3)^a^	2	2.5	2.25	2.25	1.5	1.5	1.5	1.5	0.75	0.75	0.75	0.75	0
Family leave policies total^b^ (0–9)	7	6.75	6.75	6.75	5.75	5.25	4.5	4.5	3	2.25	2.25	2.25	0

^a^ Category average score = [Leave from day 1 of hire (0–3) + Leave after day 1 of hire (0–3) + Leave after 2 years of hire (0–3) + Leave for part-time faculty (0–3)]/4. Total average category score available, 0–3.

^b^ Sum of maternal, paternal, and adoption leave averages.

Rutgers University, the University of Wisconsin, the University of Iowa, and the University of Illinois offer minimal leave reporting only Family and Medical Leave Act (FMLA) policy or FMLA plus accrued sick leave regardless of an individual's time of employment (Table 4).

### Part-time leave policies

Part-time policy scores varied widely with Michigan State University and the Ohio State University earning a score of 14 out of 15 demonstrating extremely flexible and comprehensive part-time options. Rutgers University earned a score of 1 out of 15 after a detailed review of their website found that part-time appointments were only available under certain circumstances.

Three institutions scored 12–13 out of 15 for part-time faculty policies, each offering paid maternity, paternity, and adoptive leave available to part-time faculty, as well as health benefits and part-time tenure track options (Table 5).

**Table 5.** Work-Life Policy Scores for Part-Time Faculty at Big Ten University Medical Schools

Policy: Part-time Scores	Michigan State University	Ohio State University	University of Maryland	University of Michigan	University of Minnesota	Indiana University	University of Wisconsin	University of Iowa	University of Nebraska	University of Illinois	Penn State University	Northwestern University	Rutgers University
Maternity leave for part-time faculty (0–3)	3	2	3	2	2	2	1	1	2	1	0	0	0
Paternity leave for part-time faculty (0–3)	3	3	3	3	3	3	1	1	1	1	0	0	0
Adoption leave for part-time faculty (0–3)	3	3	3	2	2	2	1	1	1	1	0	0	0
Part-time appointment (0–2)	2	2	2	2	2	1	2	2	2	1	2	1	1
Part-time tenure track (0–1)	1	1	0	1	1	0	1	1	0	1	0	0	0
Part-time health benefits (0–3)	2	3	2	2	2	1	3	2	2	2	3	2	0
**Total score (0–15)**	**14**	**14**	**13**	**12**	**12**	**9**	**9**	**8**	**8**	**7**	**5**	**3**	**1**

In comparison to the 2011 study, only 3 of 13 universities did not offer parental leave to part-time faculty. This is improved from the 2011 study when only 4 of 10 universities offered parental leave for part-time faculty.

### Part-time appointments

All institutions offered options for part-time faculty employment. This is unchanged from Welch et al.'s findings in the 2011 study.

### Part-time health benefits

All institutions offered health benefits to employees with at least 50% Full-time equivalent with the exception of Indiana University who offers health benefits to employees with greater than 75% FTE and Rutgers University who does not offer benefits to part-time employees (Table 5).

### Policies for part-time faculty tenure track

Institutions were further examined for part-time tenure track. A score of 0 indicates this is not offered and a score of 1 indicates part-time tenure is pursuable. Seven of thirteen institutions offer tenure track for part-time faculty (University of Illinois, University of Iowa, University of Michigan, Michigan State University, University of Minnesota, Ohio State University, and University of Wisconsin) (Table 5).

### Extension of probationary period

Extension of the probationary period offers further flexibility to junior faculty on the tenure track. A score of 0 indicates an institution *does not offer extension*, a score of 1 indicates a *1-year extension upon request*, and a score of 2 indicates *automatic 1-year extension*. Only three universities do not offer extension of probationary period (Penn State University, Rutgers University, and University of Maryland). The most frequently offered option was an *automatic 1-year extension* (Table 2).

### Childcare options

All institutions offered childcare options. Three schools reported affiliated childcare centers only. The remaining 10 institutions offered onsite or affiliated childcare as well as financial aid or discounts. All websites provided easy to navigate options for investigating childcare (Table 2).

### Lactation policy

Every institution reviewed offered dedicated lactation spaces, which improved from 70% of schools reporting lactation space in the 2011 study (Table 2).

### Return-to-work scheduling policy (post-partum)

Eleven of thirteen schools offer flexible scheduling options in the return-to-work or postpartum period after addition of a child. Flexible scheduling included condensed work weeks, options to work from home, and continued flexible hours to support childcare. Only the University of Iowa and Northwestern University did not offer flexible return to work scheduling options.

### Noteworthy policies falling outside study purview

During our investigation, we discovered noteworthy policies that were not captured in the scoring rubric. Northwestern University reported a robust wellness and work-life policy, in which new parents are provided consultation with a wellness team which provides guidance in managing the transition to parenthood, finding and establishing childcare, work-life integration, sends follow-up resources, and assists in application to public schools. Further, elder or senior care benefits are also offered to employees, similar to childcare benefits.

## Discussion

Our study found that overall, Big Ten Universities have updated and expanded work-life policies for their employed physicians in the past decade. The top scoring institutions in 2020 were Michigan State University and Ohio State University, followed by the University of Michigan. In comparing institutions to the 2011 study, several made strides to improve their faculty work-life policies, including University of Minnesota, University of Michigan, Michigan State, Ohio State, and Indiana University.

Flexible work-life policies improve physician wellness, reduce burnout and increase physician retention and promotion at academic institutions.^18–20^ There are multiple tangible examples of improved work-life policies that promote equity, wellness, and advancement for faculty at these medical schools. Expanding family leave to include maternity, paternity, and adoption leave supports more equitable sharing of parental responsibilities and more diverse family structures.

Part-time benefits and tenure pathways for part-time faculty are now more readily available, allowing physicians the opportunity to continue to advance their careers while spending time on activities outside of work. In addition, childcare options and flexible return-to-work scheduling ensure that key years of productivity are not lost due to childbearing, child-rearing, or other significant life events and accommodations are available for those who choose this path. Part-time options can help increase workforce diversity in other ways, such as allowing physicians to work in multiple settings while still advancing their careers in academic medicine.

Another area of improvement includes modernized lactation policies to support breastfeeding faculty members, which have resulted from an explosion of breastfeeding advocacy in the workplace and in social media.^21^ The medical field has made great strides in this area and should lead the way in supporting this healthy practice for both mothers and their infants.

Improved flexible work-life policies are vital to support both early career physicians and female physicians. In a comparative study evaluating burnout differences among American surgeons, authors found that a greater proportion of early career physicians were women and that women experience more work-home conflicts than their male counterparts. Female surgeons were more likely to believe that child-rearing had slowed their career, to have experienced a conflict with their spouse's/partner's career, and to have experienced a work-home conflict in the past 3 weeks. Women also reported significantly higher rates of burnout and depressive symptoms.^22^ Flexible work policies can help to reduce these stressors.

Return-to-work scheduling alternatives or options after the addition of a child (*e.g.*, postpartum or adoption) is another example of flexible policies to support faculty work-life choices. This is a relatively new phenomenon in the past decade. Universities that have adopted these policies recognize that the challenges of new parenthood do not disappear when parental leave ends. Return-to-work flexibility may improve infant-parent bonding, duration of breastfeeding, and parent mental health.^23,24^

It must be noted that our study was conducted during the worldwide coronavirus disease 2019 (COVID-19) pandemic. Across the country, workplaces had to reexamine their employment policies to offer more flexible work-from-home options, part-time options, and childcare options. This necessitated a review of current policies and an update that may not have occurred otherwise. Regardless of the pandemic, Big Ten institutions should be commended for offering these forward-thinking policies.

Despite advances in many of the above areas, few of the institutions that we studied expanded support for childcare during the past 10 years. We found that a common option for physician parents was a token partnership with a local center. Academic physicians require childcare that is accessible, flexible, and preferably onsite or close to campus, which can accommodate physicians' erratic and sometimes unpredictable work hours.^25^ Some institutions have responded to this need by offering financial support or discounted pricing for dependent care. Childcare is a significant stressor for academic physicians with young children and is a vital area for increased attention and future growth.^26^

Finally, our study focused solely on the existence of work-life policies at Big Ten institutions and not their implementation nor if they were accessible, affordable, or adequate. We did not study how frequently faculty members accessed the opportunities supported by these flexible work options. Others have found that academic faculty often underutilize existing policies due to absence of information, misinformation, unsupportive supervisors, or inflexible workplace cultures. To ensure that updated policies are deployed, the literature suggests that Big Ten department leaders should educate and encourage their academic physicians to utilize existing flexible work options.^11^

Furthermore, institutions with a clear, easily accessible website and human resource contact may also intentionally promote their supportive policies. This may result in more successful recruitment of talented faculty as well as retention of currently employed academic physicians.

Future studies should continue to investigate innovative categories of flexible work-life policies as well as examine their accessibility and use by faculty members.

## Limitations

Potential limitations included missing policy data and interpretation bias in reviewing the policy websites.

Most schools studied provided no centralized resource for policies. Institutions may have had policies that were available by employee-access only. Our search strategy may have not found policies that used different terminology. Baseline data were unavailable for three schools that joined the Big Ten after the 2011 study. In addition, we added a new policy category: return-to-work or postpartum preferential scheduling policies. We surmise no such category existed before the 2011 study as very few or no such policies existed.

The return-to-work policy analysis/accuracy may be limited in that many clinicians experience “local” processes that may not be a formal policy. Clinical responsibilities (return to work) are often determined at the division or department or clinic level based on coverage needs.

We recognize the need to reexamine the existence of new categories over time. A limitation of the scoring rubric is the way in which the scores are weighted. Certain audiences may ascribe more value to family leave policy than part-time policies, for example. Future studies may choose to emphasize specific policy categories over others.

Finally, conducting this study during the COVID-19 pandemic may have influenced office staff's ability to answer our requests for confirmation and clarification of policies due to absence from their physical office space.

## Conclusions

While progress has been made, every institution should challenge itself to review flexibility in work-life policies for faculty. The improvement and expansion of work-life policies must be intentional. Those institutions that scored lower in our study may consider focusing their attention to this area to better support one of their most vital assets—the highly skilled and trained physician leaders of the clinical and academic medical workforce. Specifically, Big Ten institutions can continue to advance their flexible work-life policies by providing increased access to childcare options, further expansion of parental leave (including available leave from the first day of hire), and offering more flexible options for part-time faculty.

Moreover, this scoring tool could be used across all institutions for faculty members to evaluate their own institutional policies and use these metrics to inform best practices. Healthy competition with the goal to achieve more forward-thinking policies can improve retention, recruitment, and advancement of all faculty, leading to a more productive workplace, increased diversity and inclusion, and improved overall academic physician wellness.

## Abbreviations Used

AL: adoption leave
COVID-19: coronavirus disease 2019
FMLA: Family and Medical Leave Act
FTE: Full-time equivalent
HR: Human Resource
ML: maternity leave
NCAA: National Collegiate Athletic Association
PL: paternity leave
